# Hypoxia-associated circDENND2A promotes glioma aggressiveness by sponging miR-625-5p

**DOI:** 10.1186/s11658-019-0149-x

**Published:** 2019-04-02

**Authors:** Hui Su, Defei Zou, Yikun Sun, Yiwu Dai

**Affiliations:** 1 Department of Neurosurgery, Chinese PLA General Hospital, Medical School of Chinese PLA, Beijing, People’s Republic of China; 2 Department of Neurosurgery, Bayi Brain Hospital, PLA Army General Hospital, Beijing, People’s Republic of China; 3 Department of Neurosurgery, The 306th Hospital of PLA, Beijing, People’s Republic of China

**Keywords:** circDENND2A, miR-625-5p, Glioma, Hypoxia, Migration, Invasion

## Abstract

**Background:**

As a newfound type of non-coding RNA, circular RNAs (circRNAs) are involved in various physiological and pathological processes via regulation of gene expression. Increasing evidence shows that aberrantly expressed circRNAs play a crucial role in the initiation and progression of many tumors. However, the functions of different circRNAs in gliomas remain elusive.

**Methods:**

The levels of circRNAs, miRNAs, and mRNAs were quantified by qPCR. The interaction between circDENND2A and miR-625-5p was determined by luciferase reporter and pull-down assays. The migratory and invasive capabilities of glioma cells were examined by wound healing and Transwell assays. Immunohistochemistry was performed to analyze the HIF1α level in glioma tissues.

**Results:**

We predicted circDENND2A (has_circ_0002142) to be a hypoxia-responsive circRNA in glioma via a bioinformatic analysis. We found that hypoxia induced the expression of circDENND2A, which promoted migration and invasion of glioma cells. To understand the behaviors of circDENND2A in glioma, we studied the putative miRNAs targeted by circDENND2A and identified circDENND2A as an efficient sponge of miR-625-5p in glioma cells. Phenotype experiments verified that circDENND2A was required for the hypoxia-induced migration and invasion of glioma cells and that this occurred by sponging of miR-625-5p. Notably, glioma tissues overexpressing HIF1α exhibited a high expression of circDENND2A as well as a low expression of miR-625-5p. circDENND2A was negatively correlated with miR-625-5p.

**Conclusion:**

circDENND2A is required for the hypoxia-induced malignancy of glioma cells and functions by sponging miR-625-5p.

**Electronic supplementary material:**

The online version of this article (10.1186/s11658-019-0149-x) contains supplementary material, which is available to authorized users.

## Introduction

Glioma is the most prevalent and malignant tumor of the central nervous system, originating from glial cells [1]. The highly invasive and infiltrative characteristics of glioma confer its incurability and mortality [2]. Despite many potential treatments having been applied in glioma, the outcomes are unfavorable due to this cancer’s extensive growth patterns [3]. Therefore, a detailed understanding of the mechanisms underlying glioma pathology is urgently needed for novel therapeutic approaches.

Circular RNA (circRNA) belongs to the larger category of non-coding RNA. circRNAs are characterized by covalently closed-loop structures with no 5′ to 3′ polarity and no polyadenylated tail [4]. Their circular structure confers strong resistance to RNase R and a stable existence in cells [5]. Generally, circRNA regulates gene transcription and expression by serving as a sponge of miRNA [6, 7]. Therefore, the circRNA-miRNA-mRNA regulatory axis has been widely explored to illustrate the biological functions of circRNA [8]. Increasing evidence suggests that circRNAs participate in a variety of physiological and pathological processes [9]. As critical regulators in the development and progression of several malignancies, dysregulated circRNAs promote tumor proliferation, migration, and invasion [10–12]. However, little is known about the roles of circRNAs in glioma development, especially under hypoxic conditions.

MicroRNA (miRNA) is a class of non-coding small RNAs that regulates gene expression at the post-transcriptional level [13]. Abnormal expression profiles of miRNAs have been observed in a wide range of human cancers, including glioma [14–16]. As a tumor suppressor, miR-625 is downregulated in gastric cancer, breast cancer, and hepatocellular carcinoma [17–19]. These studies indicated that the decreased expression of miR-625 was closely associated with tumor growth, invasiveness, and metastasis, and also showed that it predicted high malignancy and poor prognosis. The levels of miR-625 have been consistently found to be inversely correlated with glioma grades. miR-625 inhibits the proliferation and increases the chemosensitivity of glioma cells [20]. However, the reason behind miR-625’s effects on glioma is still unknown.

In the present study, we found that hsa_circ_0002142 (circDENND2A), a circRNA derived from the *DENND2A* gene, was induced by hypoxia in glioma cells, and the circDENND2A possessed a potent ability to sponge miR-625-5p. Phenotype experiments indicated that the circDENND2A was indispensable for the hypoxia-induced migration and invasion of glioma cells by sponging miR-625-5p. Furthermore, clinical analysis confirmed the negative correlation between circDENND2A and miR-625-5p in glioma tissues.

## Materials and methods

### Cell culture and treatment

The normal human astrocyte line SVGp12 and two human glioma cell lines, U87MG and A172, were purchased from American Type Culture Collection (ATCC, Manassas, VA, USA) and maintained in Dulbecco’s Modified Eagle’s medium (DMEM) with 10% fetal bovine serum (Gibco, Waltham, MA, USA) at 37 °C under an atmosphere of 5% CO_2_. In hypoxia experiments, cells were cultured in 1% O_2_, 5% CO_2_, and 94% N_2_. Plasmids, miRNA, and small interfering RNAs (siRNAs) were transfected into U87MG cells with Lipofectamine™ 3000 Transfection Reagent (Invitrogen, Carlsbad, CA, USA) according to the manufacturer’s protocol.

### Constructions and reagents

CircDENND2A overexpression plasmid and siRNA, miR-625-5p mimic, and inhibitor were synthesized by GenePharma (Shanghai, China). The sequences of circDENND2A siRNA, miR-625-5p mimic, and inhibitor are shown in Additional file 1: Table S1.

### Quantitative real-time PCR (qPCR) for circRNA, mRNA, and miRNA

Total RNA was extracted using the RNAiso™ Plus reagent (Takara, Otsu, Japan). For RNase R digestion, 5 mg total RNA was treated for 15 min at 37 °C with 5 U/mg RNase R. Then, qPCR for circRNA and mRNA was performed with SYBR Green PCR Master Mix (Applied Biosystems, Foster City, CA, USA). miRNA-specific quantitative PCR was carried out with TaqMan MicroRNA Assay primers (Applied Biosystems). circRNA/mRNA and miRNA were individually normalized to β-actin and U6. The primer sequences of the circRNAs and mRNAs are shown in Additional file 1: Table S1.

### Fluorescence in situ hybridization (FISH)

DNA oligo probes labeled with FITC for circDENND2A were synthesized by GenePharma (Shanghai, China). U87MG cells in the logarithmic phase were washed with PBS and fixed in 4% paraformaldehyde. After permeabilization with PBS containing 0.5% Triton X-100 for 5 min at 4 °C, the cells were incubated in hybridization buffer (40% formamide, 10% dextran sulfate, 1 × Denhardt’s solution, 4 × SSC, 10 mM DDT, 1 mg/mL yeast transfer RNA, and 1 mg/mL sheared salmon sperm DNA) with the probes specific to circDENND2A at 42 °C overnight. Nuclei were stained with 4, 6-diamidino-2-phenylindole (DAPI). Images were acquired using a fluorescence microscope.

### Wound healing assay

U87MG cells were seeded in six-well plates. After transfection for 24 h, the monolayer was gently scratched with a pipette tip and then washed with phosphate-buffered saline (PBS) to remove detached cells. Each well was re-loaded with serum-free medium, and cells were cultured for additional 48 h, when images of the scratched monolayers were captured on a microscope. The wounds were evaluated using ImageJ.

### Transwell assay

The invasive capability of U87MG cells was evaluated in Boyden chambers with Matrigel according to the manufacturer’s protocol (Invitrogen). The 8-mm-porosity polycarbonate membrane was covered with 200 mL of serum-free medium containing 1 × 10^5^ cells per well. The plates were then incubated with 10% fetal bovine serum (FBS) medium for 48 h at 37 °C in a 5% CO_2_ incubator. The invading cells on the bottom surface of the filter were fixed, stained, and counted using optical microscopy.

### Pull-down assay

U87MG cells were transfected with biotinylated miR-625-5p mimics or control miRNA and harvested at 48 h after transfection. The biotin-labeled miRNA was pulled down by incubating the cell lysates with Dynabeads™ M-270 Streptavidin Beads (Invitrogen). The bound RNA was purified using a PureLink™ RNA Mini Kit (Invitrogen) for analysis. The abundance of circDENND2A was evaluated by qPCR analysis.

### Luciferase reporter assay

pGL3-circDENND2A was constructed based on pGL3-control, and pGL3-circDENND2A with a mutant miR-625-5p binding site was subcloned from wild-type pGL3-circDENND2A. U87MG cells were plated onto 24-well plates the day before transfection. The cells were co-transfected with 0.5 μg of wild-type or mutant pGL3-circDENND2A, 0.02 μg of pRL-TK Renilla luciferase reporter plasmids (Promega, Madison, WI, USA), and miR-625-5p mimics. Luciferase activity was measured by a Dual-Luciferase Reporter Assay System (Promega).

### Immunohistochemistry (IHC)

In total, 180 glioma cases came from the National Human Genetic Resources Sharing Service Platform (No. 2005DKA21300), and informed consent was obtained from all of the patients. The study was approved by the Ethics Committee of Chinese PLA General Hospital and Shanghai Outdo Biotech Company (Shanghai, China). All of the specimens were fixed in 10% neutral formalin, embedded in paraffin, and cut into 4-μm sections for immunohistochemical staining. The EnVision™ two-step method was used (DAKO, Hamburg, Germany), along with an antibody against HIF1a. To estimate the score for each slide, at least 10 individual fields at 200× were chosen, and 100 cancer cells were counted in each field. The immunostaining intensity was divided into four grades: 0, no expression; 1, mildly positive; 2, moderately positive; and 3, markedly positive. The proportion of positive-staining cells was divided into five grades: 0, < 10%; 1, 11–25%; 2, 26–50%; 3, 51–75%; and 4, > 75%. The staining results were assessed and confirmed by two independent investigators blinded to the clinical data. The percentage of positivity of the tumor cells and the staining intensities were then multiplied in order to generate the IHC score, and graded as 0–6, low expression; and 7–12, high expression.

### Statistical analysis

Statistical data analysis was performed using SPSS 22.0 and GraphPad Prism 5.0. The data are reported as the mean ± SD. A *P* value < 0.05 was considered statistically significant. The two-tailed Student’s t test and analysis of variance (ANOVA) were used to determine significant differences. A χ2 test was used to compare the clinicopathological features of glioma patients with HIF1a expression. The Pearson test was used in analyzing the correlations.

## Results

### CircCENND2A is a hypoxia-responsive circRNA in glioma cells

**Fig. 1:**
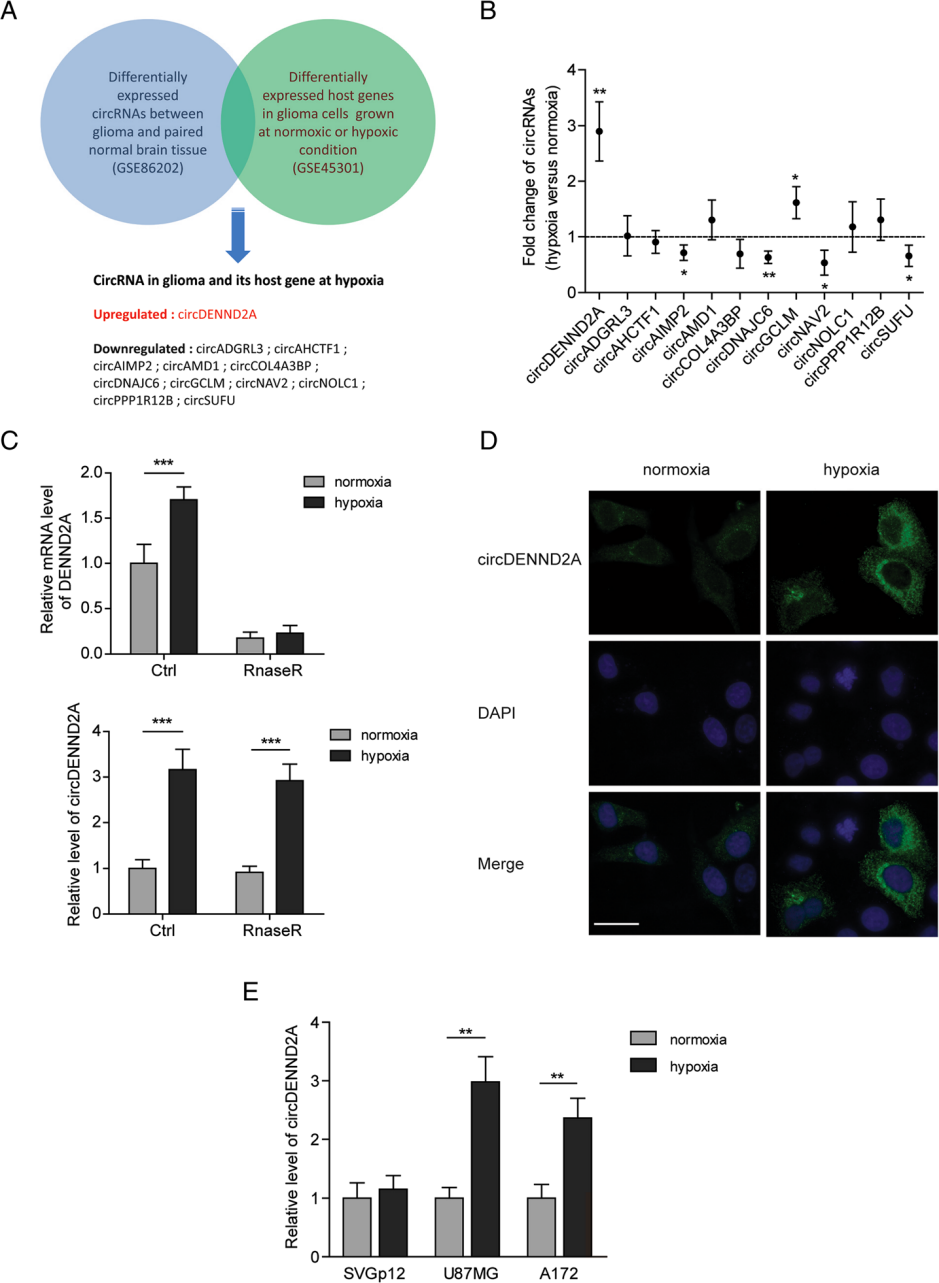
CircCENND2A is a hypoxia-responsive circRNA in glioma cells. **a** Hypoxia-associated circRNAs in glioma were screened by comparing the differentially expressed circRNA profiles between glioma and normal tissues (GSE86202) and by comparing the differentially expressed mRNA profiles in U87MG cells between hypoxia and normoxia (GSE45301). **b** Fold changes of the screened circRNAs under hypoxia versus normoxia were measured by qPCR in U87MG cells. **c** The levels of DENND2A mRNA or circRNA in normoxic or hypoxic U87MG cells treated with or without RNase R were measured by qPCR. **d** RNA fluorescence in situ hybridization showed the abundance and localization of circDENND2A in normoxic or hypoxic U87MG cells. DAPI solution was used to stain the nuclei. **e** The levels of DENND2A circRNA in normoxic or hypoxic SVGp12, U87MG, and A172 cells were measured by qPCR. Data are shown as mean ± SD (three independent repeats). * *p* < 0.05, ** *p* < 0.01, *** *p* < 0.001

In order to profile the hypoxia-associated circRNAs, we compared the differentially expressed circRNA profiles between glioma and normal tissues (GSE86202) [21] with the differentially expressed mRNA profiles in U87MG cells between hypoxia and normoxia (GSE45301) [22]. As shown in Fig. 1a, expression changes of 12 circRNAs in glioma tissues and their host genes in hypoxia were confirmed, suggesting potential hypoxia-associated circRNAs in glioma. Notably, the expression levels of only one circRNA (circDENND2A) and its host gene (DENND2A) were found to be upregulated. To identify the circRNAs involved in response to hypoxia, we examined the levels of the 12 circRNAs in hypoxic or normoxic U87MG cells. Two circRNAs, most noticeably circDENND2A, were significantly increased, and four circRNAs showed decreases in the U87MG cells treated with hypoxia (Fig. 1b). We found that the hypoxia-induced DENND2A circRNA but not mRNA was resistant to RNase R exonuclease, which verified that circDENND2A was induced by hypoxia (Fig. 1c). In addition, fluorescence in situ hybridization (FISH) also indicated the hypoxia-induced accumulation of circDENND2A in the cytoplasm of U87MG cells (Fig. 1d). Notably, the expression of circDENND2A was induced by hypoxia in two glioma cell lines, U87MG and A172, but not in the normal human astrocyte line SVGp12 (Fig. 1e).

### CircDENND2A contributes to the hypoxia-induced migration and invasion of glioma cells

**Fig. 2:**
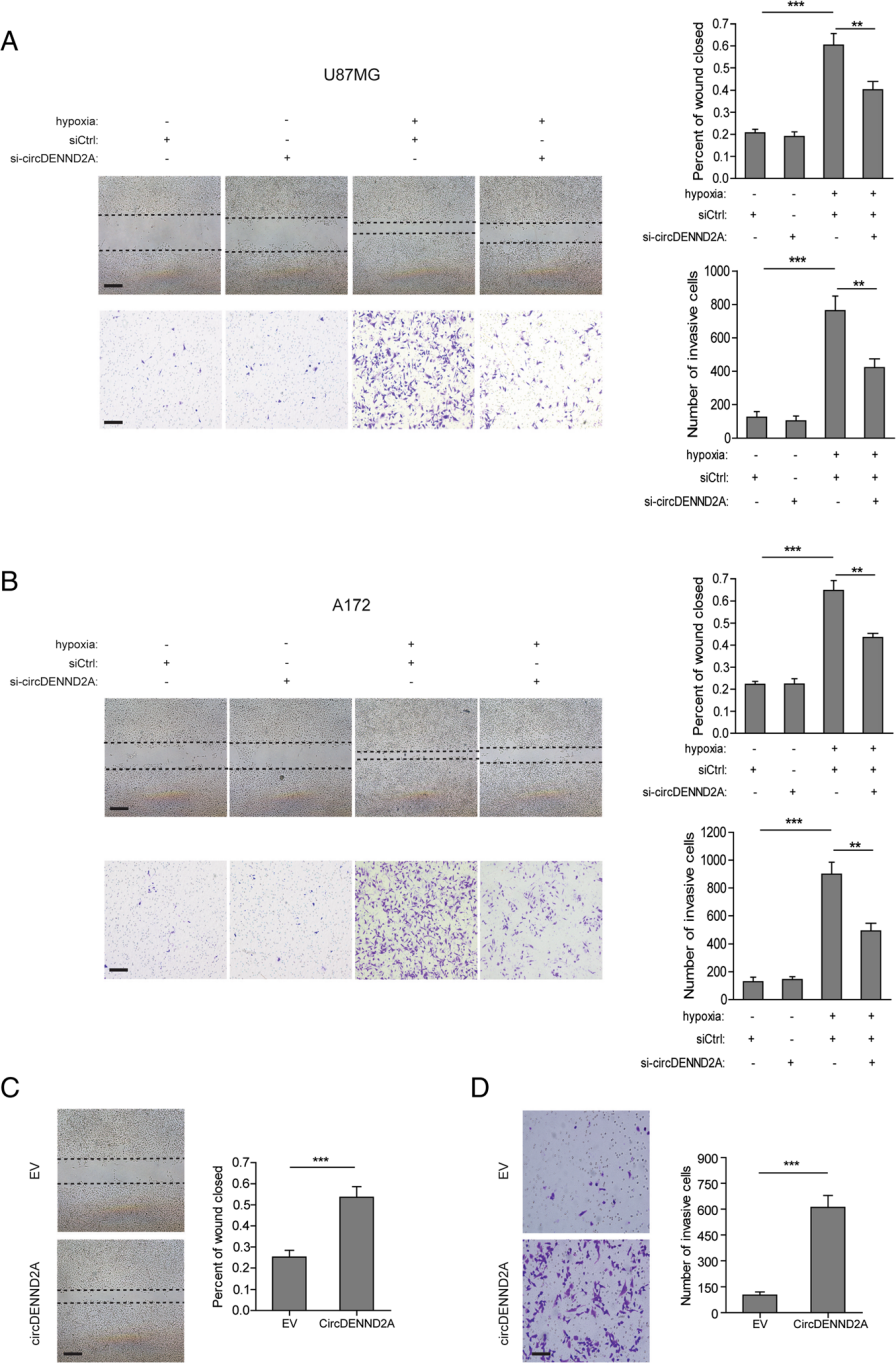
CircDENND2A contributes to the hypoxia-induced migration and invasion of glioma cells. **a** and **b** Left: Representative images from wound healing assays or Transwell assays in normoxic or hypoxic U87MG (**a**) and A172 (**b**) cells with or without knockdown of circDENND2A. Scale bar, 100 μm. Right: Percentage of wound closure or numbers of invasive cells at 48 h after treatment. **c** and **d** Left: Representative images from wound healing assays or Transwell assays in U87MG cells with or without overexpression of circDENND2A. Scale bar, 100 μm. Right: Percentage of wound closure or numbers of invasive cells at 48 h after treatment. Data are shown as mean ± SD (three independent repeats). * *p* < 0.05, ** *p* < 0.01, *** *p* < 0.001

To further reveal the role of circDENND2A in glioma development, we examined the phenotype of glioma cells with knockdown or overexpression of circDENND2A. The results of wound healing and Transwell assays showed that hypoxia promoted the migration and invasion of U87MG and A172 cells, while knockdown of circDENND2A undermined the hypoxia-induced aggressive characteristics (Fig. 2a and b). In contrast, knockdown of circDENND2A had no effect on the phenotypes of glioma cells under normoxic conditions (Fig. 2a and b). Like hypoxia, overexpression of circDENND2A strengthened the migratory and invasive capabilities of U87MGcells (Fig. 2c and d).

### CircDENND2A functions as an efficient miR-625-5p sponge in glioma cells

**Fig. 3:**
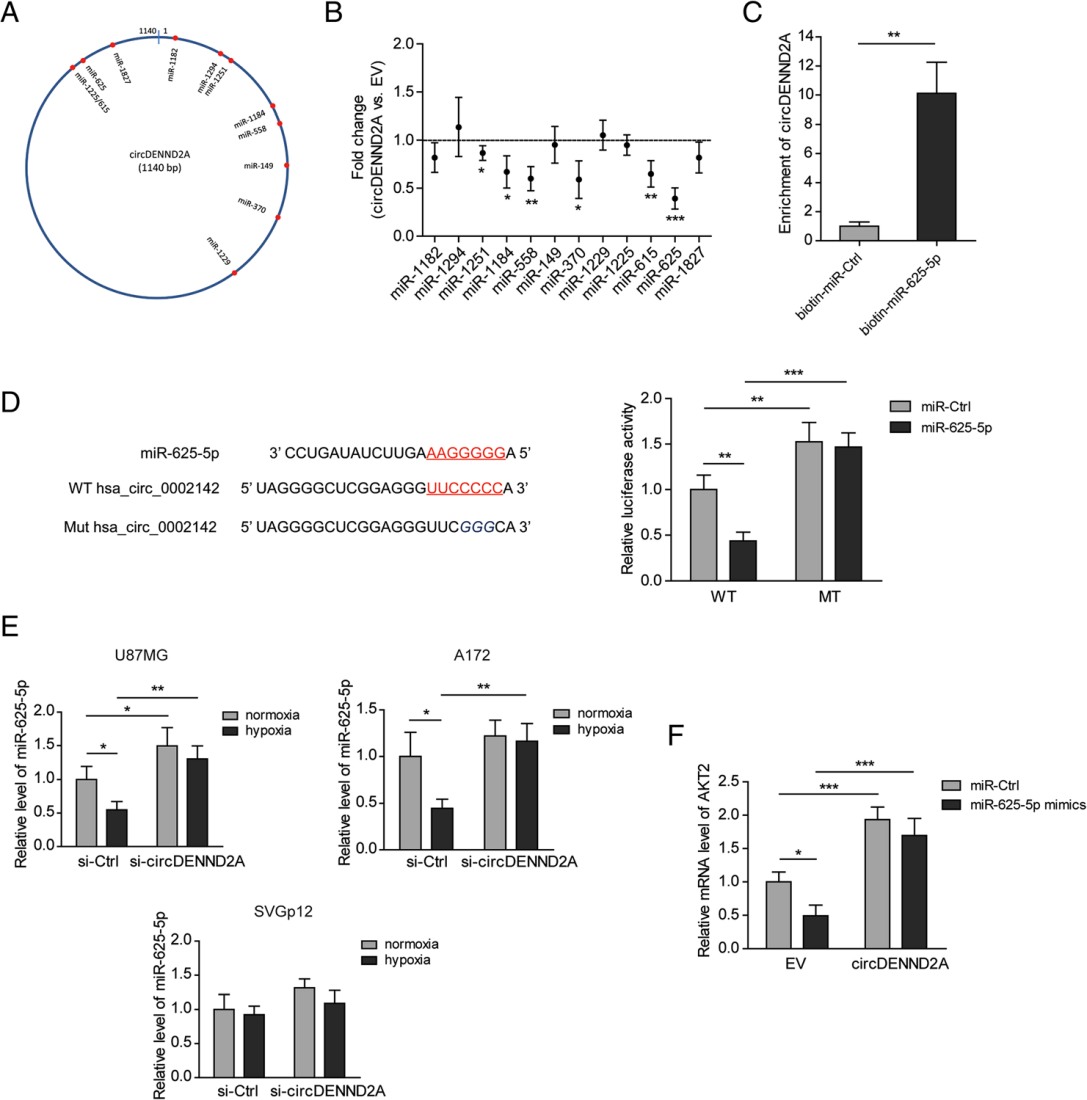
CircDENND2A functions as an efficient miR-625-5p sponge in glioma cells. **a** Schematic drawing showing the putative binding sites for miRNAs in circDENND2A. **b** Fold changes of the 12 miRNAs in U87MG cells with overexpression of circDENND2A versus empty vector (EV) were determined by qPCR. **c** The levels of circDENND2A captured by biotinylated miR-625-5p or miR-Ctrl from U87MG cell lysates were measured by qPCR. **d** The luciferase activities of wild-type or mutant pGL3-control-circDENND2A in U87MG cells transfected with miR-625-5p or miR-Ctrl. **e** The levels of miR-625-5p in hypoxic or normoxic U87MG, A172, and SVGp12 cells with or without knockdown of circDENND2A were measured by qPCR. **f** The mRNA levels of AKT2 in U87MG cells transfected with circDENND2A or EV and miR-625-5p or miR-Ctrl were determined by qPCR. Data are shown as mean ± SD (three independent repeats). * *p* < 0.05, ** *p* < 0.01, *** *p* < 0.001

To explore the mechanisms underlying the positive effect of circDENND2A on glioma progression, we predicted the putative miRNAs sponged by circDENND2A via Circular RNA Interactome (https://circinteractome.nia.nih.gov/). Eleven miRNAs were screened out based on the context + score percentile (> 90) (Fig. 3a). After cells were transfected with circDENND2A, six miRNAs were significantly decreased, among which the most obvious one was miR-625-5p (Fig. 3b). Furthermore, pull-down assays indicated a much stronger enrichment of circDENND2A in the biotin-labeled miR-625-5p-captured fraction than in the negative control (Fig. 3c). A luciferase reporter assay showed that miR-625-5p suppressed the activity of a reporter plasmid containing wild-type circDENND2A but failed to do so when a circDENND2A plasmid with a mutant miR-625-5p-binding site was used. This suggested that there is an interaction between wild-type circDENND2A and miR-625-5p (Fig. 3d). Hypoxia reduced the level of miR-625-5p in U87MG and A172 cells, but this miRNA was increased by knockdown of circDENND2A. However, neither hypoxia nor circDENND2A knockdown altered the level of miR-625-5p in SVGp12 cells (Fig. 3e). As a validated target of miR-625-5p [20], AKT2 was inhibited by miR-625-5p transfection. However, overexpressed circDENND2A blocked the effect of miR-625-5p (Fig. 3f). In summary, circDENND2A can regulate the expression levels of some downstream genes by sponging miR-625-5p.

### CircDENND2A enhances the migration and invasion of glioma cells via sponging of miR-625-5p

**Fig. 4:**
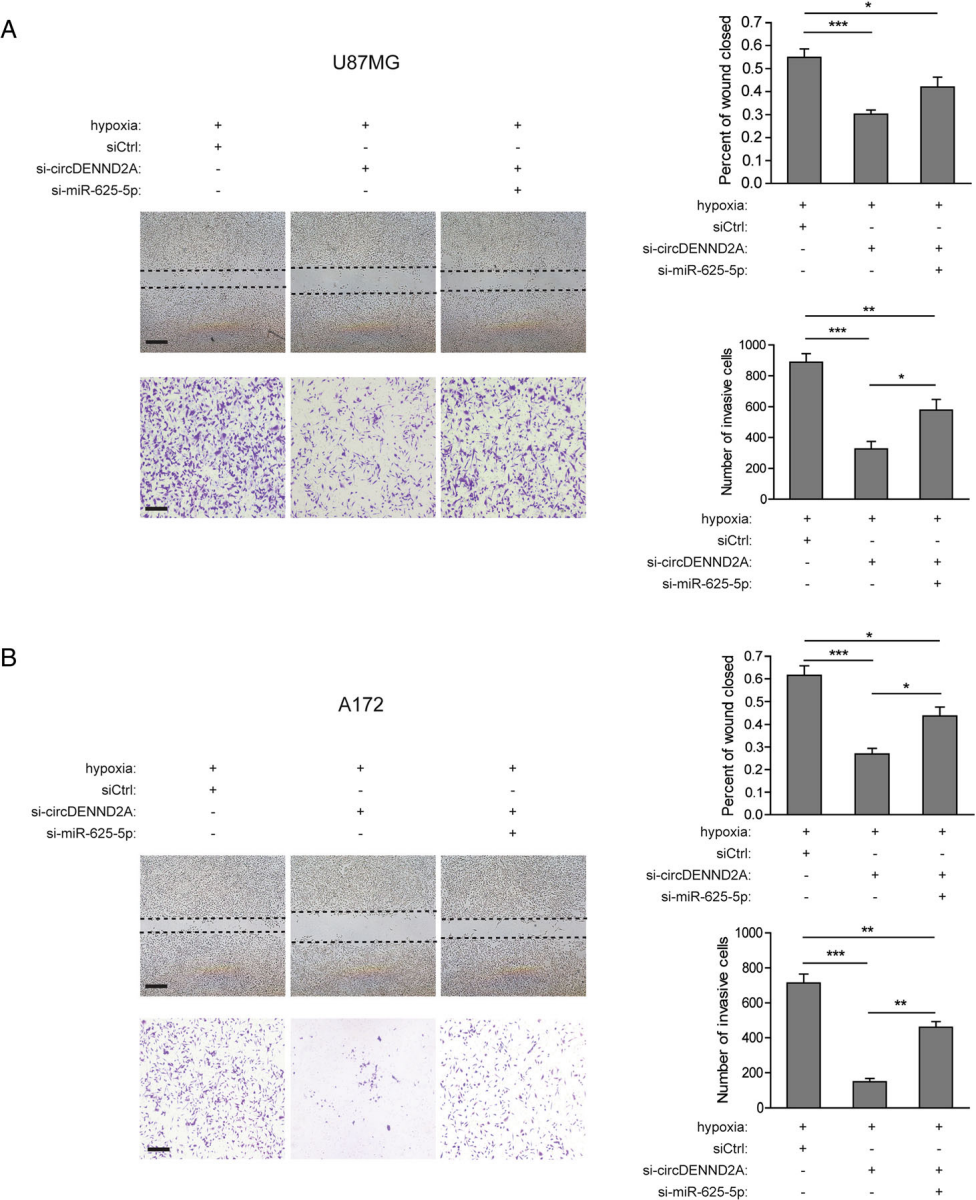
CircDENND2A enhances the migration and invasion of glioma cells via sponging of miR-625-5p. **a** and **b** Left: Representative images from wound healing assays or Transwell assays in hypoxic U87MG and A172 cells with or without knockdown of circDENND2A or miR-625-5p. Scale bar, 100 μm. Right: Percentage of wound closure or numbers of invasive cells at 48 h after treatment. Data are shown as mean ± SD (three independent repeats). * *p* < 0.05, ** *p* < 0.01, *** *p* < 0.001

To determine whether miR-625-5p is involved in the malignancy driven by circDENND2A, we studied the phenotypes of glioma cells with knockdown of circDENND2A or miR-625-5p. As shown in Fig. 4a and b, silencing of miR-625-5p restored the hypoxia-induced migration and invasion attenuated by circDENND2A knockdown in U87MG and A172 cells. Therefore, circDENND2A may act as a potent inhibitor of the tumor suppressor miR-625-5p to promote glioma progression.

### The circDENND2A/miR-625-5p pathway is related to HIF1α in glioma tissues

**Fig. 5:**
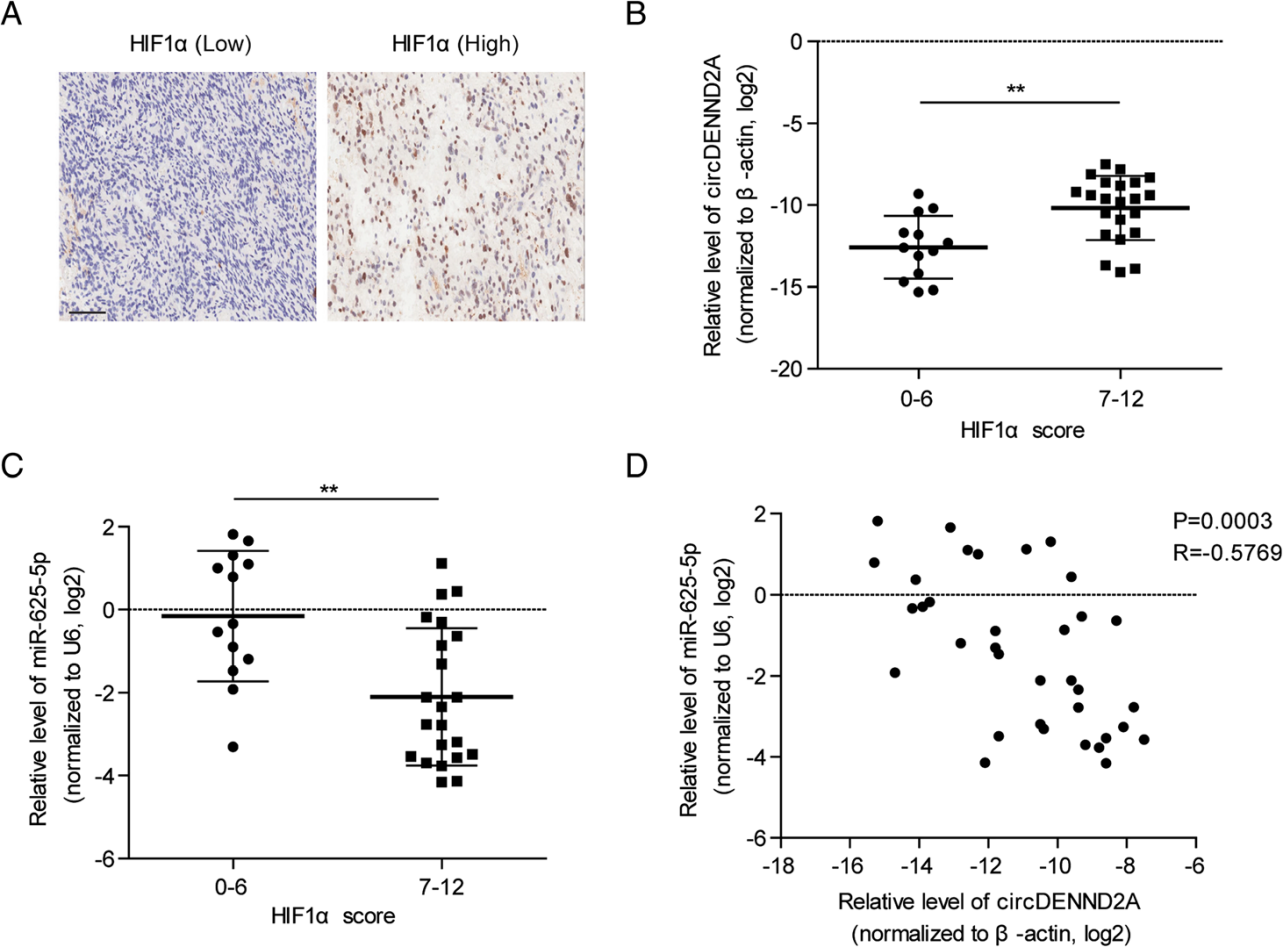
The circDENND2A/miR-625-5p pathway is related to HIF1α in glioma tissues. **a** Representative IHC images of glioma tissues with high or low expression of HIF1α. **b and c** Based on the IHC score of HIF1α, 35 glioma tissues were separated into a high-HIF1α expression group (IHC score: 0–6, *n* = 22) and a low-expression group (IHC score: 7–12, *n* = 13). The relative levels of circDENND2A (**b**) or miR-625-5p (**c**) in the high- or low-expression group were quantified by qPCR and then individually normalized to β-actin and U6. **d** Correlation between circDENND2A and miR-625-5p in the 35 glioma tissues. ** *p* < 0.01

Overexpression of HIF1α leads to tumor progression and poor prognosis in patients with glioma. In this study, an analysis of clinical relevance indicated that the levels of HIF1α were positively associated with WHO grades in 180 glioma cases (Table 1), which was consistent with previous reports [23]. HIF1α is a specific marker for hypoxia. We analyzed the relation between HIF1α and circDENND2A or miR-625-5p. With immunohistochemistry, 35 frozen glioma tissues from the 180 cases were separated into a high HIF1α-expression group (IHC score: 0–6) and a low-expression group (IHC score: 7–12) (Fig. 5a). The high-expression group exhibited a higher level of circDENND2A but a lower level of miR-625-5p than the low-expression group (Fig. 5b and c). Further analysis indicated a negative correlation between circDENND2A and miR-625-5p in glioma (Fig. 5d).

**Table 1:** Correlation of the expression of HIF1a with clinicopathological features in glioma

	Total	HIF1a		
		low	high	*P*
	180	91	89	
Gender				0.5897
male	112	55	57	
female	68	36	32	
Age				0.1287
< 40	80	43	37	
≥ 40	100	48	52	
Tumor location				0.9403
Temporal	56	26	30	
Frontal	54	28	26	
Parietal	12	5	7	
Ventricle	11	6	5	
Occipital	9	5	4	
Others	38	21	17	
WHO grade				< 0.0001*
I	26	14	12	
II	85	54	31	
III	42	20	22	
IV	25	3	22	
Unknown	2	0	2	

*P < 0.05

## Discussion

Glioma is characterized by extensive tissue hypoxia due to intratumoral necrosis. A hypoxic microenvironment has been thought to be a driving force in glioma, which develops an aggressive tumor phenotype by displaying high levels of metastasis and losing apoptotic potential [24]. Recent studies have described hypoxia as an essential environmental condition for the maintenance of glioma stem cells (GSCs), the subpopulation of glioma cells leading to resistance to chemotherapy and radiation [25]. As circRNA has attracted more attention in recent years, several circRNAs have been demonstrated in the development of glioma [26]. However, the interaction between hypoxia and circRNA in glioma remains unknown. Previous work first identified hypoxia-regulated circRNAs in endothelial cells [27]. On the basis of this discovery, Yang et al. found that the circRNA cZNF292 contributed to glioma tube formation via the Wnt/β-catenin signaling pathway [28]. Here, we have discovered a hypoxia-induced circRNA, circDENND2A, by analyzing the profiles of differentially-expressed circRNAs in glioma tissues and mRNAs in hypoxic glioma cells. Further investigation confirmed that the upregulated circDENND2A was necessary for increased migration and invasion of hypoxic glioma cells. Therefore, we hypothesized that circDENND2A may display a pivotal role in the glioma metastasis induced by hypoxia.

Hypoxia-inducible factor 1α (HIF1α) is a hallmark of hypoxia. It activates the transcription of downstream oncogenes containing hypoxia-responsive elements (HREs), thereby regulating tumor metabolism and metastasis [29]. Overexpression of HIF1α in surgically resected glioma tissues has been found to have a positive correlation with invasiveness and poor survival [30]. In the present study, we explored the association between HIF1α and circDENND2A or miR-625-5p. Glioma tissues with overexpressed HIF1α showed high levels of circDENND2A and low levels of miR-625-5p. Liang et al. reported that in breast cancer cells, HIF1α was crucial for the upregulation of circDENND4C under hypoxic conditions [31]. However, the crosstalk between HIF1α and the circDENND2A/miR-625-5p pathway in glioma still needs more investigation.

In this work, circDENND2A was shown to enhance the migration and invasion of glioma cells by sponging miR-625-5p, indicating that miR-625-5p served as a tumor suppressor in glioma. The mechanisms underlying the anti-tumor function of miR-625-5p in different cancer types had been demonstrated in previous studies. In breast cancer, miR-625-5p suppressed cell proliferation and migration by targeting HMGA1 [18]. In another report, miR-625-5p targeted IGF2BP1 to inhibit the migration and invasion of hepatocellular carcinoma [19]. Zhang et al. found that miR-625-5p decreased the proliferation and increased the chemosensitivity of glioma via AKT2 [20]. Notably, SOX2 has been validated as a target of miR-625, as it induced migration and invasion in melanoma and esophageal cancer [32, 33]. As an embryonic transcription factor, SOX2 maintains cancer stem cells and drives the epithelial-to-mesenchymal transition (EMT) [34]. Thus, it will be worthwhile to investigate whether the circDENND2A/miR-625-5p pathway participates in hypoxia-induced stemness and EMT in glioma by regulating SOX2.

In conclusion, our study characterizes a novel hypoxia-associated circRNA, circDENND2A, which enhances the migration and invasion of glioma cells by directly sponging miR-625-5p. Clinical analysis suggested the existence of a circDENND2A/miR-625-5p axis in glioma tissues, which was associated with HIF1a. Collectively, understanding the mechanism of this interaction may provide us a promising therapeutic target for glioma metastases in hypoxic microenvironments.

## Additional file

Additional file 1: **Table S1.** The sequences of circDENND2A siRNA, miR-625-5p mimic and inhibitor. (DOCX 17 kb)

## Abbreviations

circRNA: Circular RNA
EMT: Epithelial-to-mesenchymal transition
GSCs: Glioma stem cells
HIF1a: Hypoxia-inducible factor 1a
HRE: Hypoxia-responsive element
miRNA: MicroRNA
