# Supplementary material for: Hypoxia-associated circDENND2A promotes glioma aggressiveness by sponging miR-625-5p
Source: Cell Mol Biol Lett. 2019 Apr 2;24:24. doi: 10.1186/s11658-019-0149-x (PMC6446274; doi:10.1186/s11658-019-0149-x)
Supplement: Supplementary file 1 — Table S1. The sequences of circDENND2A siRNA, miR-625-5p mimic and inhibitor. (DOCX 17 kb) [file 11658_2019_149_MOESM1_ESM.docx]

**Table S1** The sequences of circDENND2A siRNA, miR-625-5p mimic and inhibitor.

|  | **Name** | **Sequence(5'-3')** |  |
| --- | --- | --- | --- |
| oligo RNAs | circDENND2A-siRNA | UUGACCCUGUGGUCUGCACUG |  |
|  | miR-625-5p mimic | AGGGGGAAAGUUCUAUAGUCC |  |
|  | miR-625-5p inhibitor | GGACUAUAGAACUUUCCCCCU |  |
|  |  | **Forward Sequence(5'-3')** | **Reverse Sequence(5'-3')** |
| oligo DNAs (Primers) | circDENND2A(hsa_circ_0002142) | TGAACAGAAGACTGTGGACCG | CAGTCTCTAGGAATGGAATGGAGG |
|  | circADGRL3 | CGGCTCCTGACAACAAATAAGAC | AGCCAGCTCTCTAGCAATGT |
|  | circAHCTF1(hsa_circ_0017331) | AGCCATTGAACGAGCTAAGCC | TGCCTCCTTCATCTCGTTTCC |
|  | circAIMP2(hsa_circ_0134468) | AGAGCGTGCCTGAAAACCTT | TCATCTTGGCGGGACTCAAG |
|  | circAMD1(hsa_circ_0005954) | AAGGGTACCCACACCGGAA | CATAAGCTTCCTGCTTGTCAGT |
|  | circCOL4A3BP(hsa_circ_0073050) | AATCCCACTTTGGAGGACCAG | GTGGATGTTGCAGAGTAGCC |
|  | circDNAJC6(hsa_circ_0002454) | CCAGACATCTTGACCACTACACA | TGTCAAAGAGACCTCCCCCA |
|  | circGCLM(hsa_circ_0003513) | TAATCTTGCCTCCTGCTGTGT | TTCTACTGCATGAGATACAGTGC |
|  | circNAV2(hsa_circ_0095567) | GGGAGGGATCGACAAGCAAG | AAGTCGGTGAACCACACTCTC |
|  | circNOLC1(hsa_circ_0000257) | ATTCTGACTCAAGCTCCGAGG | GGAAGAGGCATTGGCATCCT |
|  | circPPP1R12B(hsa_circ_0002414) | CCTCTGACCTTGCAGAAGAGC | TGTCTTGCTGGTTTACATTGGC |
|  | circSUFU(hsa_circ_0003779) | TGTCCTTCCACCAATCAACCC | AGCAGCATGTGCTGAATTCTTG |
|  | DENND2A | AACTGAAGGCCATTCCCCAG | TCTTCGGCAGTAACCGAACC |
|  | AKT2 | TCCGAGGTCGACACAAGGTA | CTGGCCGAGTAGGAGAACTG |
|  | β-actin | ATCATTGCTCCTCCTGAGCG | ACTCCTGCTTGCTGATCCAC |
